# The Area Prostriata may play a role in technical reasoning

**DOI:** 10.1186/s12993-022-00200-9

**Published:** 2022-11-25

**Authors:** Giovanni Federico, Carlo Cavaliere, Emanuelle Reynaud, Marco Salvatore, Maria Antonella Brandimonte, François Osiurak

**Affiliations:** 1 IRCCS SYNLAB SDN S.p.A, Via Emanuele Gianturco, 113, 80143 Naples, Italy; 2 Laboratoire d’Etude des Mécanismes Cognitifs (EA 3082), Université de Lyon, Lyon, France; 3 Laboratory of Experimental Psychology, Suor Orsola Benincasa University, Naples, Italy; 4 Institut Universitaire de France, Paris, France

## Abstract

Most recent research indicated how technical reasoning (TR), namely, a specific form of causal reasoning aimed at understanding the physical world, may support the development of tools and technologies of increasing complexity. We have recently identified the Area PF of the left inferior parietal lobe (PF) as a critical structural correlate of TR, as assessed by using two ad-hoc psycho-technical tests evaluating the two main aspects of TR, i.e., physical world’s understanding and visuospatial imagery. Here, we extended our findings by implementing new ad-hoc analyses of our previous data by using a whole-brain approach. Results showed that the cortical thickness (CT) of the left Area Prostriata of the visual cortex, alongside the left Area PF CT, predicts TR performance.

## Main

**Fig. 1:**
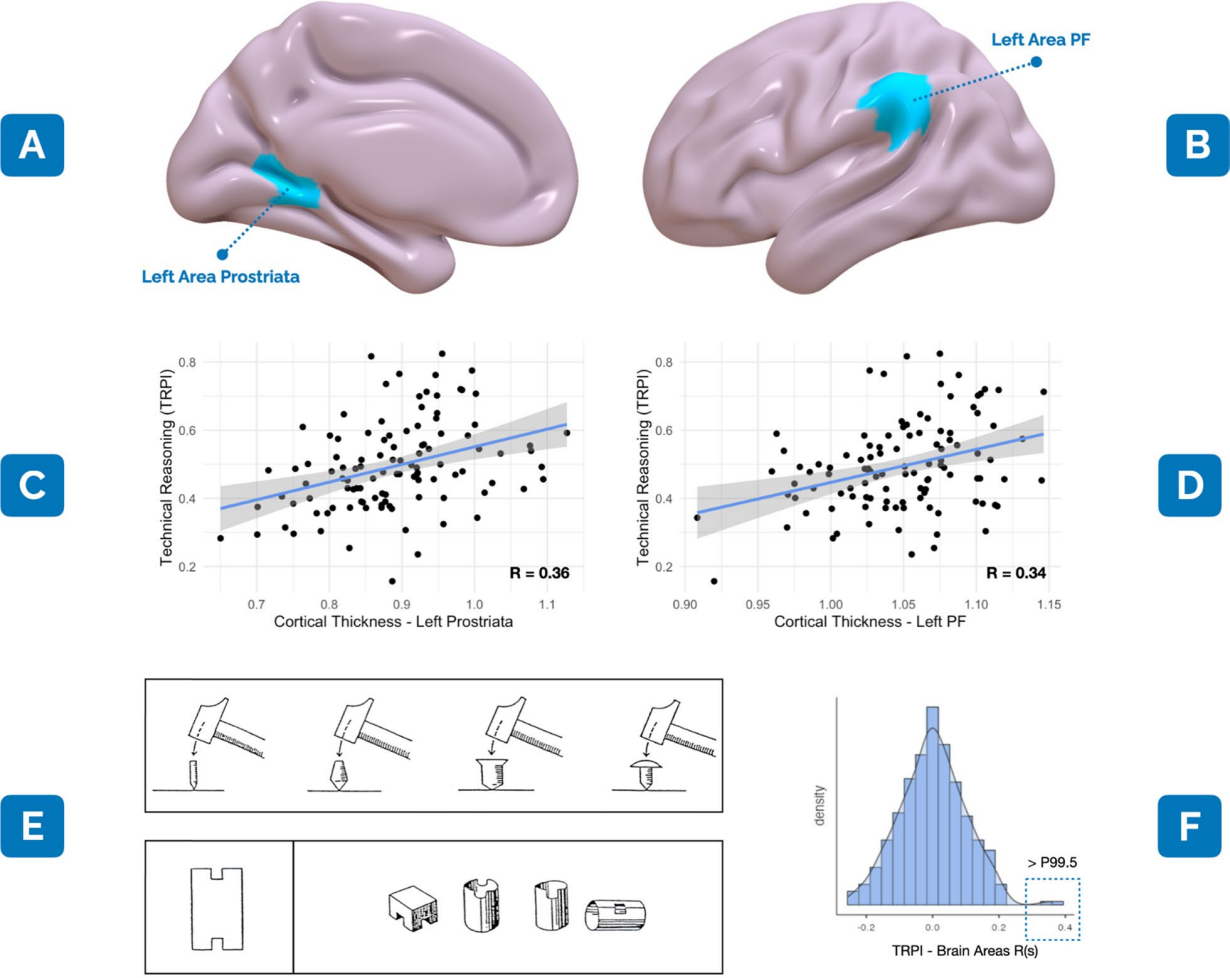
The left Area Prostriata and Technical Reasoning. **A** The left Area Prostriata (in light blue), as included in Glasser et al. (2016)’s atlas [16]. **B** In light blue, the area PF of the left Inferior Parietal Lobe (IPL), as included in Glasser et al. (2016)’s atlas [16]. **C** Pearson’s correlation between the technical reasoning performance index (TRPI) [5] and the normalised cortical thickness (CT) of the Area Prostriata (R = 0.36). **D** Pearson’s correlation between TRPI and the CT of the area PF (R = 0.34). **E** Above is an example of the 24 items we used in Federico et al. (2022) [17] to evaluate the understanding of physical properties (e.g., participants were asked to select which of the four nails were hammered more easily). Below is an example of the 38 items aimed at evaluating participants’ visuospatial skills (e.g., to identify which of the four 3D figures shown on the right corresponds to the bi-dimensional pattern on the left). Both the subtests were extracted from the NV7 battery (https://www.pearsonclinical.fr/nv7). **F** The normal distribution (density) of Pearson’s correlations between TRPI [17] and the CT of each brain area that is included in Glasser et al. (2016)’s atlas [16]

The Area Prostriata of the visual cortex (AP) and technical reasoning (TR, i.e., a specific form of causal reasoning aimed at understanding the physical world) have both been neglected by cognitive neuroscientists [1–3]. Indeed, while the first functional characterisation of such a “new” visual system was proposed by Mikellidou and colleagues in 2017 [4], only recently TR have entered the neuroscientific debate as a human-characterising cognitive process that enabled the evolution of tools and technologies [2, 3]. Recently, we have identified the cortical thickness (CT), i.e., a brain-volume-related measure linked to cognitive performance, of the Area PF of the left inferior parietal lobe (Area PF; Fig. 1B) as a critical structural correlate of TR [5]. Here, we extend our findings by performing a whole-brain analysis of our previous data. We found that the CT of the left AP (Fig. 1C), along with the left Area PF CT (Fig. 1D), mediates TR skills.

AP is a small occipital lobe region described by Sanides in 1969 [6] that is located in the medial wall of the calcarine sulcus, surrounded by the retrosplenial and parahippocampal cortices, anteriorly, and by the far peripheral representation of V1 and ventral V2, posteriorly. Despite its spatial contiguity with V1, AP resembles many features of limbic cortices and evolutionary-ancient structures, namely, the “Prokoniocortex” cytoarchitectural pattern, which consists of small and densely packed layer-4 cells characterised by a thinner layer 4 and a thicker layer 2 [1]. AP’s structural connectivity has been investigated in animal models, but limited findings have been reported using diffusion tensor magnetic resonance imaging in the human brain [7]. AP’s afferents are distinct multimodal cortical areas such as the primary and secondary visual (i.e., V1 and V2) and auditory (i.e., A1 and A2) cortices, limbic structures (e.g., rhinal cortex and subiculum) and several subcortical regions, such as the anterior and midline thalamic nuclei and claustrum. AP receives direct projections from the rostral part of the dorsal lateral geniculate nucleus (dLGN). The dLGN-AP pathway may have a key role in the AP’s functional specialisation, that is, blindsight and fast processing of information from the far peripheral visual fields, particularly for fast-moving objects [1, 4]. Specifically, such a pathway seems to include two subcomponents that pass to the optic radiations ventrally and dorsally. These subcomponents appear to be specifically involved in peripheral and central visual-field representations. The functional retinotopic parcellation of AP is supported by eccentricity, myelin and CT gradients, as well as by gene expression studies [8].

AP outputs are multisensory and high-order association cortical areas (e.g., V1, the contralateral AP, and the temporal, parietal, anterior cingulate, orbitofrontal, and frontopolar cortices) and subcortical regions linked to the visuomotor function and visuospatial abilities (e.g., the subiculum, pulvinar, ventral lateral geniculate nucleus, lateral dorsal thalamic nucleus, zona incerta, and the pontine and pretectal nuclei) [4, 7, 8]. Visuospatial abilities and the visuomotor function are at the root of TR [5, 9]. Indeed, most recent research emphasises how TR can be seen as a cognitive process emerging by adaptation from visuospatial skills [5]. TR has been defined as a specific form of non-declarative knowledge about physical principles that enables individuals to develop and use complex tools, techniques and technologies [2, 5, 9]. Such knowledge can be abstract because physical and technical realities do not always overlap. For instance, a single physical matter (e.g., glass) can have multiple properties (e.g., transparency, hardness and sharpness). Contrariwise, distinct physical matters (e.g., plastic or metal) can have the same single property (e.g., hardness). TR, like other types of reasoning, is causal, allowing one to anticipate the outcomes of future physical events. However, it is also analogical, allowing individuals to transfer their understanding from one situation to another [3].

Identifying the neural and cognitive bases that underlie the complexity of human tools and technologies over generations, namely, the cumulative technological culture (CTC), has been considered one of the millennium’s most essential questions [10]. Nevertheless, the neuroscientific literature has started to consider TR as a cognitive process directly involved in CTC only a few years ago [3, 11]. Research on TR identified the involvement of multiple brain regions belonging to distinct left-lateralized networks (i.e., tool-use and action-observation networks) [12, 13]. Within these networks, most recent studies have reported the specificity of the left inferior frontal gyrus (IFG) and the Area PF of the left IPL in TR (Fig. 1B). Congruently, brain-lesion investigations demonstrated deficits in using familiar and novel tools (i.e., TR-related tasks) after damage to the left Area PF [14]. Along with the left frontoparietal involvement in TR, increasing evidence have detailed how the simulation of physical events activates imagery-like representations, which recruit visual areas [15]. Thus, when individuals predict the trajectory of a falling ball, they recruit occipital motion-sensitive brain regions, even when no motion is being sensed. Therefore, on the one hand, TR involves frontoparietal areas related to the physical world’s understanding. On the other hand, more posterior regions are involved in generating mental simulations of actions, which enable individuals to make predictions about the outcomes of physical scenes.

The structural contribution of occipital regions in TR has never been explored in the literature. In structural imaging, CT is a measure that reflects the size, density and arrangement of cells in a brain region. Differences in CT of multiple brain regions have been correlated with the performance of distinct cognitive processes [5]. In a recent study, we have found that the CT of the left Area PF predicts TR performance on psycho-technical tests in which right-handed participants (N = 116; 70 females; mean age = 23.9 years, SD = 3.9) solved physical and visuospatial problems [5]. These tests evaluated the two TR key aspects, i.e., the physical world’s understanding and visuospatial imagery (see Fig. 1E for details). As the study’s neuroanatomical focus was on the IPL, we selected the CT of all regions of the left and right IPL as potential predictors of TR, identifying the CT of the left PF as the only significant IPL predictor of TR.

Here, we aim to extend previous results by using a whole-brain approach. We, therefore, re-analysed our previous data by calculating Pearson’s correlations between the CT of each participant’s brain area (i.e., 360 regions [16]) and the TR performance index we devised {TRPI; see Federico et al. (2022)’s Methods for details [5]}. As a first exploratory analytical approach, in the normal distribution of correlations we obtained (Shapiro-Wilk W = 0.99, Shapiro-Wilk p = 0.29; Fig. 1F), we identified as positive correlations of interest (CoI) only the ones that, in our sample, were above the right 99.5th percentile. In so doing, we obtained only two CoI: the first between TRPI and the left PF (R = 0.34; P99.7; p < 0.01; Fig. 1D); the second between TRPI and the left AP (R = 0.36; P99.9; p < 0.01; Fig. 1C). Then, to identify an appropriate predictive model and to correct for multiple comparisons, we implemented a 10-fold cross-validated stepwise forward analysis which included TRPI as the dependent variable and the CT of each brain area as potential predictors (min = 1; max = 20 predictors). The cross-validation indicated the CT of left Area PF and left AP, taken together, as the biggest weighted predictors of TRPI (R^2^ = 0.27, RMSE = 0.12; *nvmax* = 1:20). Multiple regression was used to test whether the CT of these regions significantly predicted TRPI. The regression explained 24% of the variance [R^2^ = 0.24, F(2, 105) = 16.33, p < 0.001].

The results presented here extend our previous findings concerning the structural neural correlates of TR by suggesting a potential key role of the left AP in the technical mind’s genesis. The contribution of posterior areas in TR adds to what is already known about the wide fronto-temporo-parietal network involved in integrating semantic, technical and sensorimotor knowledge to understand the physical world [9, 17, 18]. However, while the idea of a contribution of the visual system to the simulation of physical events is not a new concept in cognitive neuroscience [15], how posterior regions may take part in integrating and manipulating technical contents through which individuals can decode physical events remains essentially undiscovered. As discussed above, TR originates from visuospatial skills, although it is not entirely coincident with them [3, 5]. We share visuospatial abilities with other species and, congruently, AP is a conserved region that is found in rodents and primates. Therefore, rather than imagining an exclusive contribution of AP in TR, it is reasonable to assume that such an area may participate in visuospatial-related cognitive processes that together make up the capabilities of the human technical mind. Thus, such a multisensory primary area may account for a much more fundamental role on the top of which TR can operate. While future functional investigations are certainly necessary to detail the visual system’s role in TR, the present results may hopefully provide the impetus for new research into this unexplored region of the visual system.

## Data Availability

The data supporting the present study’s findings are available at https://osf.io/thu74.
